# The efficacy of fall-risk-increasing drug (FRID) withdrawal for the prevention of falls and fall-related complications: protocol for a systematic review and meta-analysis

**DOI:** 10.1186/s13643-017-0426-6

**Published:** 2017-02-20

**Authors:** Justin Yusen Lee, Anne Holbrook

**Affiliations:** 1 Division of Geriatric Medicine, Department of Medicine, McMaster University, Hamilton Health Sciences, 88 Maplewood Avenue, Hamilton, ON L8M 1W9 Canada; 2 Division of Clinical Pharmacology and Toxicology, Department of Medicine, McMaster University, St. Joseph’s Healthcare Hamilton, 50 Charlton Avenue East, Hamilton, Ontario L8N 4A6 Canada

**Keywords:** Falls, Falls prevention, Fall-risk increasing drug (FRID), Deprescribing, Medication withdrawal, Systematic review

## Abstract

**Background:**

Despite limited evidence of effectiveness, withdrawal (discontinuation or dose reduction) of high risk medications known as “fall-risk increasing drugs” (FRIDs) is typically conducted as a fall prevention strategy based on presumptive benefit. Our objective is to determine the efficacy of fall-risk increasing drugs (FRIDs) withdrawal on the prevention of falls and fall-related complications.

**Methods/design:**

We will search for all published and unpublished randomized controlled trials evaluating the effect of FRID withdrawal compared to usual care on the rate of falls, incidence of falls, fall-related injuries, fall-related fractures, fall-related hospitalizations, or adverse effects related to the intervention in adults aged 65 years or older. Electronic database searches will be conducted in MEDLINE, EMBASE, Cochrane Central Register of Controlled Trials (CENTRAL), and CINAHL. A grey literature search will be conducted including clinical trial registries and conference proceedings and abstracts. Two reviewers will independently perform in duplicate citation screening, full-text review, data abstraction, and risk of bias assessment. Conflicts will be resolved through team discussion or by a third reviewer if no consensus can be reached. The Grades of Recommendation, Assessment, Development and Evaluation (GRADE) criteria will be used to independently rate overall confidence in effect estimates for each outcome. Results will be synthesized descriptively, and a random effects meta-analysis will be conducted for each outcome if studies are deemed similar methodologically, clinically, and statistically.

**Discussion:**

We will attempt to determine whether a FRID withdrawal strategy alone is effective at preventing falls in older adults. Our results will be used to optimize and focus fall prevention strategies and initiatives internationally with a goal of improving the health of older adults.

**Systematic review registration:**

PROSPERO CRD42016040203

**Electronic supplementary material:**

The online version of this article (doi:10.1186/s13643-017-0426-6) contains supplementary material, which is available to authorized users.

## Background

Falls and fall-related injuries among older adults are significant public health concerns due to their high incidence and associated morbidity and mortality. Every year, 1 in 3 seniors aged 65 years or older fall and 10% of these falls cause serious injury and/or hospitalization [1]. Current fall prevention clinical practice guidelines focus on multi-component assessment and intervention strategies [2]. However, it is unclear which parts of the multi-component strategy are effective and how large the treatment effect is for individual interventions.

Despite limited evidence of effectiveness, withdrawal (discontinuation or dose reduction) of high risk medications known as “fall-risk increasing drugs” (FRIDs) is typically included in these multi-component strategies. The justification for FRID withdrawal is based on retrospective observational data showing that the use of certain medications is a significant risk factor for falls. These medications include anti-hypertensives (e.g., diuretics, beta-blockers), anti-arrhythmics, anti-cholinergics, anti-histamines, sedatives and hypnotics (e.g., benzodiazepines), neuroleptics, antidepressants, narcotics, and nonsteroidal anti-inflammatory drugs (NSAIDs) [3–5]. This evidence, however, is based primarily on observational data with minimal adjustment for confounders, dosage, or duration of therapy. It is therefore unclear whether the associated increase in falls is truly related to the use of these drugs or the underlying conditions that the drugs are treating.

In order to justify the current common practice of FRID withdrawal, the presumption of its effectiveness as a falls prevention strategy needs to be confirmed. A prospective cohort study showed that FRID withdrawal was associated with a reduction in falls (HR 0.48, 95% CI 0.23–0.99) [6], but this needs to be replicated with high quality randomized controlled trials (RCT) evidence. There have been a number of systematic reviews that have assessed the effectiveness of all available interventions designed to prevent falls [7–10]. However, only two of these systematic reviews were designed specifically for medication-related interventions for falls prevention, and their methodological quality is relatively low (AMSTAR scores of 3) [1, 11]. To date, there is no systematic review that has specifically examined the effectiveness of FRID withdrawal on falls prevention.

The most recent systematic review with strong methodological rigour is a 2012 Cochrane review of fall prevention interventions in the community [7]. It identified five trials investigating the effect of medication withdrawal. Gradual withdrawal of psychotropic medications in one RCT reduced the rate of falls (RaR 0.34, 95% CI 0.16–0.73), but not the risk of falling [12]. Three of four RCTs of medication review and modification did not reduce falling rate or risk.

Our specific systematic review research question is “In older adults age ≥65 years, does the withdrawal of fall-risk increasing drugs (FRIDs) decrease the risk of falls compared to usual care and continuation of these drugs?”. The aim of this systematic review is to assess the effectiveness of FRID withdrawal as an individual intervention to prevent falls across all care settings and to clarify the evidence base supporting its current practice and presumptive effectiveness. It will update previous systematic reviews by incorporating new recent data from the largest RCT of FRID withdrawal to date [13], focus future medication-related fall prevention strategies, and identify current gaps in evidence.

## Methods/design

This protocol was developed using the Cochrane Handbook for Systematic Reviews of Interventions as methodological framework and reported in accordance with the Preferred Reporting Items for Systematic Reviews and Meta-Analyses Protocols (PRISMA-P) guidelines [14, 15]. A completed PRISMA-P recommendation checklist is included as an additional file (see Additional file 1). Our protocol has been registered in the PROSPERO database (CRD42016040203).

## Eligibility criteria

### Types of studies

All published and unpublished RCTs, cluster RCTs, and quasi-RCTs (e.g., allocation by alternation or date of birth) comparing a FRID withdrawal intervention (discontinuation or dose reduction) to usual care (i.e., no withdrawal) will be included.

### Types of participants

We will include all studies focused on older adults aged 65 years or older from all settings (e.g., community, acute care, long-term care, and rehabilitation).

### Types of interventions and comparators

We will include all interventions that withdraw FRIDs (single or multiple medications) with the intent of reducing the risk of future falls compared to usual care (i.e., no FRID withdrawal and/or no change in usual activities). The intervention may be accompanied by a preceding medication review for FRID withdrawal appropriateness. There will be no restrictions placed on the professional background of the individual conducting FRID withdrawal and/or medication review. Studies involving FRID withdrawal as part of a more complex multi-component intervention will be excluded.

### Types of outcome measures

The primary outcomes of this review are the (1) rate of falls and (2) incidence of falls (i.e., number of fallers). Secondary outcomes will include the incidence of (1) fall-related fractures, (2) fall-related injuries, (3) fall-related hospitalization, (4) adverse effects related to the withdrawal intervention (e.g., disease relapse, symptomatic withdrawal).

## Search methods

Comprehensive search strategies will be developed in consultation with an experienced librarian. Electronic database searches will be conducted in MEDLINE, EMBASE, Cochrane Central Register of Controlled Trials (CENTRAL), and Cumulative Index to Nursing and Allied Health Literature (CINAHL) using a combination of medical subject headings, controlled and free-text terms with various synonyms for the intervention. The Ovid Medline search strategy is shown as an example in an additional file (see Additional file 2). A similar search strategy will be used in the other databases. No restrictions will be placed on language, year of publication, or time (i.e., studies of all durations will be included).

The search will be repeated prior to final manuscript submission. The reference lists of all included studies, relevant systematic reviews, and guidelines will be manually hand searched for additional eligible studies. We will perform a grey literature search through the following: (1) trial registries (e.g., ClinicalTrials.gov, World Health Organization Clinical Trials Search Portal) and (2) conference proceedings and abstracts in for the most recent meetings (2012 to 2015) of the Canadian Geriatrics Society, American Geriatrics Society, and European Union Geriatric Medicine Society. We will contact primary authors to determine whether results are available and include these trials in the systematic review if appropriate.

## Study selection

Two independent reviewers will screen available titles and abstracts to assess for possible inclusion and full-text review using the Covidence online software (https://www.covidence.org). From the full text, two reviewers will independently assess potentially eligible trials in duplicate for inclusion. Studies will be included when both reviewers agree about their inclusion. Any disagreement will be resolved through discussion or by a third reviewer if no consensus can be reached. If needed, authors will be contacted for any additional details required to clarify study eligibility. Reason(s) for exclusion of any studies will be documented. Eligible study citations will be saved into the Mendeley reference manager library.

In an initial pilot run of study selection of a random sample of 50 citations, the kappa statistic will be measured to determine inter-rater agreement on study selection and reveal any problems with the protocol requiring modification. Full screening will only begin when there is >95% agreement.

## Data extraction

Study data will be extracted independently in duplicate by two reviewers using a data extraction form developed in Microsoft Word. The following information will be extracted: study characteristics, patient characteristics, intervention characteristics, and outcome results from each time period reported in the study and/or the longest duration of follow-up. Any disagreement will be resolved through discussion or by a third reviewer if no consensus can be reached. Study authors will be consulted for additional or missing information where appropriate. All reviewers will pilot test the data extraction form prior to the full review to identify any problems with the form requiring modification.

## Assessment of risk of bias

All included studies will be assessed in duplicate by two independent reviewers for risk of bias using a modified version of the Cochrane Risk of Bias tool based on random sequence generation (selection bias), allocation concealment (selection bias), blinding of participants, personnel and outcome assessors (performance and detection bias), completeness of follow-up (attrition bias), selective outcome reporting, and other biases (e.g., recall bias due to unreliable method of falls ascertainment) [16]. Each criterion will be assigned a score of *definitely low risk*, *probably low risk*, *probably high risk*, or *definitely high risk* [17]. *Definitely* and *probably* scores will be collapsed to *low risk* and *high risk*, respectively, to allow for low vs. high risk sensitivity analysis. Any disagreement will be resolved through discussion or by a third reviewer if no consensus can be reached.

Selective outcome reporting bias will be examined by making efforts to access study protocols (e.g., obtain published protocols, check trial databases, or request from study author). Outcomes reported in the protocol will be compared with those reported in the paper. Furthermore, outcomes reported in the methods will be compared with those reported in the results sections of the study papers.

If there are ten or more included studies in the meta-analysis for one of the outcomes, a funnel plot will be constructed to visually inspect for potential publication bias.

## Measurement of treatment effects

The treatment effect for rate of falls will be reported as a rate ratio (RaR) and 95% confidence interval (CI) between the intervention and comparator groups. The rate of falls will be defined as the total number of falls per unit of person time that falls were monitored (e.g., falls per person year). If both adjusted and unadjusted rate ratios are reported, the unadjusted estimate will be used unless adjustment was conducted to account for cluster design effect. If a rate ratio is not reported, the rate ratio and 95% CI will be calculated using raw data (if available) of the total number of falls, and the actual total length of time falls were monitored (person years).

The treatment effect for the dichotomous incidence outcomes (i.e., incidences of falls, fall-related fracture, fall-related injury, fall-related hospitalization, and adverse effects related to the withdrawal intervention) will be reported as a risk ratio (RR) and 95% CI. If both adjusted and unadjusted risk ratios are reported, the unadjusted estimate will be used unless adjustment was conducted to account for cluster design effect. If a risk ratio is not reported or an odds ratio is reported, the risk ratio and 95% CI will be calculated using raw data (if available).

For any included cluster RCTs where treatment effects are reported at the cluster rather than the individual level, adjustments for the cluster design effect using intra-cluster coefficient (ICC) estimates and average cluster size will be conducted (if available).

## Data synthesis and summary of results

A GRADE (Grades of Recommendation, Assessment, Development and Evaluation) evidence profile (EP) and summary of findings (SoF) table will be created for each outcome [18]. Prior to conducting meta-analysis, we will assess for statistical, clinical, and methodological heterogeneity. Statistical analysis will be conducted using RevMan 5.1 and SPSS Statistics v20. If deemed feasible and appropriate, meta-analysis will be conducted. Since variations in methodological, participant, and medication characteristics are expected between studies, a random effects model will be used. Data will be pooled to estimate the treatment effect at various time intervals depending on the follow-up duration of the included studies (i.e., <3 months, 3–6 months, 6–12 months, ≥12 months). Pooled data will be presented using forest plots and qualitative descriptions. For studies where meta-analysis is not appropriate, results will be described qualitatively, and their significance to the rest of the body of evidence will be discussed.

## Assessment of heterogeneity

Heterogeneity within a pooled group of trials will be assessed using a combination of visual inspection of the forest plot and consideration of statistical tests for heterogeneity. The presence of statistically significant heterogeneity will be measured by calculating the Chi square statistic, and the degree of heterogeneity will be quantified by calculating the *I* ^2^ statistic. The *I* ^2^ will be interpreted using the thresholds described by the Cochrane Collaboration [14]. A two-tailed test with *p* value < 0.10 will be considered to be significant for all analyses.

If substantial statistical heterogeneity is found (e.g., *I* ^2^ ≥ 50%) or substantial clinical or methodology heterogeneity is noted (based on team’s expertise), sources of heterogeneity will be explored via a priori subgroup analyses. We are proposing five a priori hypotheses to explain possible heterogeneity between studies (see Additional file 3). These include differences in baseline propensity for falls as influenced by (1) a history of recurrent falls (e.g., known faller or not) or (2) place of residence or care (e.g., community, long-term care); differences in the intervention as influenced by (3) specific medication class(es) chosen for withdrawal and (4) preceding medication review by clinician for FRID withdrawal appropriateness; as well as differences in methodology based on (5) definitions used for “falls” (e.g., observed vs. self-reported).

## Management of missing data

Any missing outcome data will be requested from authors. If missing data still exists, a senior methodologist will be consulted to advise whether it is appropriate to impute missing data with replacement values (e.g., last observation carried forward). Otherwise, analysis will be conducted on the final available data, and the potential impact of missing data on the review findings will be addressed in the Discussion section of the final report.

## Sensitivity analyses

Sensitivity analyses will be conducted to compare results with (1) low vs. high risk of bias studies and (2) intention-to-treat vs. per-protocol analysis.

## Assessment of confidence in estimates of effects

Two independent reviewers will assess in duplicate the confidence in the estimates of the effects of the intervention (i.e., quality of evidence) for each reported outcome using the GRADE approach [18]. Confidence will be rated as *high*, *moderate*, *low*, or *very low* for each of the following criteria: risk of bias, inconsistency, indirectness, imprecision, and publication bias. Any disagreement will be resolved through discussion or by a third reviewer if no consensus can be reached.

## Discussion

Understanding and advancing our knowledge of the most effective fall prevention strategies is crucial to improving the health of older adults. Falls and their associated complications can cause significant morbidity, mortality and burden on patients, their families, and society. In Canada, for example, falls are the leading cause of injury and injury-related hospitalizations for seniors in Canada with annual healthcare costs exceeding $2 billion.

Medication optimization and FRID withdrawal has commonly been included as part of multi-component fall prevention interventions based on lower quality, retrospective evidence of association. Given the significant time and resources often required to successfully deprescribe patients from many of these medications, it is imperative to confirm its presumptive effectiveness as a falls prevention strategy.

To our knowledge, this will be the first systematic review evaluating the efficacy and safety of FRID withdrawal on the prevention of falls and fall-related complications in older adults. Our review will use high quality, rigorous systematic review methodology including an independent duplicate risk of bias assessment and an assessment of the quality of evidence using the GRADE approach. The results of the systematic will be reported in accordance with the PRISMA statement [19].

We will ensure that our results will be used to inform the activities of a wide variety of knowledge users and stakeholders including patients, clinicians, administrators, and policy-makers. Our findings will be disseminated through a variety of end-of-grant knowledge translation strategies including conference presentations, peer-reviewed publications, and dissemination to relevant stakeholder groups with an interest in the management and prevention of falls.

## Additional files

Additional file 1: PRISMA-P (Preferred Reporting Items for Systematic review and Meta-Analysis Protocols) 2015 checklist: recommended items to address in a systematic review protocol. (PDF 535 kb)
Additional file 2: OVID MEDLINE Search Strategy. (PDF 203 kb)
Additional file 3: Potential sources of heterogeneity. (PDF 189 kb)

## Abbreviations

AMSTAR: A Measurement Tool to Assess Systematic Reviews
CENTRAL: Cochrane Central Register of Controlled Trials
CI: Confidence interval
CINAHL: Cumulative Index to Nursing and Allied Health Literature
GRADE: Grades of Recommendation, Assessment, Development and Evaluation
ICC: Intra-cluster coefficient (ICC)
NSAID: Nonsteroidal anti-inflammatory drugs
PRISMA: Preferred Reporting Items for Systematic Reviews and Meta-Analyses
RaR: Rate ratio
RCT: Randomized controlled trial
RR: Risk ratio
