# Supplementary material for: The efficacy of fall-risk-increasing drug (FRID) withdrawal for the prevention of falls and fall-related complications: protocol for a systematic review and meta-analysis
Source: Syst Rev. 2017 Feb 20;6:33. doi: 10.1186/s13643-017-0426-6 (PMC5319057; doi:10.1186/s13643-017-0426-6)
Supplement: Additional file 2: — OVID MEDLINE Search Strategy. (PDF 203 kb) [file 13643_2017_426_MOESM2_ESM.pdf]

Additional File 2: Example of search strategy in Ovid MEDLINE

Ovid MEDLINE(R) In-Process & Other Non-Indexed Citations, Ovid MEDLINE(R) Daily and Ovid MEDLINE(R) 1946 to Present

#	Searches
1	exp Accidental Falls/pc [Prevention & Control]
2	fall.mp. [mp=title, abstract, original title, name of substance word, subject heading word, keyword heading word, protocol supplementary concept word, rare disease supplementary concept word, unique identifier]
3	falls.mp. [mp=title, abstract, original title, name of substance word, subject heading word, keyword heading word, protocol supplementary concept word, rare disease supplementary concept word, unique identifier]
4	exp Deprescriptions/
5	((medicat* or drug*) adj3 (deprescib* or withdraw* or cessat* or stop* or discontin*)).mp. [mp=title, abstract, original title, name of substance word, subject heading word, keyword heading word, protocol supplementary concept word, rare disease supplementary concept word, unique identifier]
6	((antihypertensive* or diuretic* or beta-blocker* or sedative* or hypnotic* or neuroleptic* or antipsychotic* or antidepressant* or benzodiazepine* or narcotic* or opioid* or narcotic* or NSAID*) adj3 (deprescrib* or withdraw* or cessat* or stop* or discontin*)).mp. [mp=title, abstract, original title, name of substance word, subject heading word, keyword heading word, protocol supplementary concept word, rare disease supplementary concept word, unique identifier]
7	fall-risk increasing drugs.mp.
8	FRID.mp.
9	((medicat* or drug*) adj3 (review* or improv* or program*)).mp. [mp=title, abstract, original title, name of substance word, subject heading word, keyword heading word, protocol supplementary concept word, rare disease supplementary concept word, unique identifier]
10	exp "Drug-Related Side Effects and Adverse Reactions"/pc [Prevention & Control]
11	exp Medication Therapy Management/ or exp "Drug Utilization Review"/
12	4 or 5 or 6 or 7 or 8 or 9 or 10 or 11
13	1 or 2 or 3
14	12 and 13
15	remove duplicates from 14
16	exp Clinical Trial/
17	(randomized or randomised).ab,ti.
18	placebo.ab,ti.
19	randomly.ab,ti.

20	groups.ab,ti.
21	randomized controlled trial.pt.
22	controlled clinical trial.pt.
23	16 or 17 or 18 or 19 or 20 or 21 or 22
24	15 and 23
