# Supplementary material for: The efficacy of fall-risk-increasing drug (FRID) withdrawal for the prevention of falls and fall-related complications: protocol for a systematic review and meta-analysis
Source: Syst Rev. 2017 Feb 20;6:33. doi: 10.1186/s13643-017-0426-6 (PMC5319057; doi:10.1186/s13643-017-0426-6)
Supplement: Additional file 3: — Potential sources of heterogeneity. (PDF 189 kb) [file 13643_2017_426_MOESM3_ESM.pdf]

Additional File 3: Potential Sources of Heterogeneity

Study Factor	Potential Source of Heterogeneity	Hypothesized Treatment Effect Modification
**Patient**	History of falls (e.g. known faller vs. non-faller)	Fallers will have higher baseline risk and therefore greater absolute benefit from intervention.
	Place of residence or care (e.g. community, acute care, long-term care)	Participants in institutionalized settings will have higher baseline risk and therefore greater absolute benefit from intervention.
**Intervention**	Specific medication class(es) chosen for withdrawal (e.g. antipsychotic vs. sedatives)	Withdrawal of certain medication classes may cause greater absolute benefit from intervention [6].
	Conduct of preceding medication review by clinician for FRID withdrawal appropriateness	Preceding medication review may increase likelihood of benefit and decrease likelihood of adverse effects from the intervention [7, 8].
**Outcome**	Timing of outcome measurement (e.g. 30 d vs. 1 year follow-up period)	Risk of falling may increase during acute period of medication modification, but treatment effect more benefit over time [10].
**Methodology**	Definitions used for "falls" (e.g. observed vs. self-reported)	Self-reported falls may show greater absolute benefit due to recall bias.
	Degree of successful FRID withdrawal (e.g. dose reduction only vs. complete withdrawal)	Complete withdrawal may show greater absolute benefit of intervention
